# *Phytophthora sojae* Effector PsCRN70 Suppresses Plant Defenses in *Nicotiana benthamiana*

**DOI:** 10.1371/journal.pone.0098114

**Published:** 2014-05-23

**Authors:** Nasir Ahmed Rajput, Meixiang Zhang, Yanyan Ru, Tingli Liu, Jing Xu, Li Liu, Joseph Juma Mafurah, Daolong Dou

**Affiliations:** Department of Plant Pathology, Nanjing Agricultural University, Nanjing, China; Zhejiang University, China

## Abstract

*Phytophthora sojae*, an oomycete pathogen, produces a large number of effector proteins that enter into host cells. The Crinklers (Crinkling and Necrosis, CRN) are cytoplasmic effectors that are conserved in oomycete pathogens and their encoding genes are highly expressed at the infective stages in *P. sojae*. However, their roles in pathogenesis are largely unknown. Here, we functionally characterized an effector *PsCRN70* by transiently and stably overexpressing it in *Nicotiana benthamiana*. We demonstrated that PsCRN70 was localized to the plant cell nucleus and suppressed cell death elicited by all the tested cell death-inducing proteins, including BAX, PsAvh241, PsCRN63, PsojNIP and R3a/Avr3a. Overexpression of the *PsCRN70* gene in *N. benthamiana* enhanced susceptibility to *P. parasitica*. The H_2_O_2_ accumulation in the *PsCRN70*-transgenic plants was reduced compared to the *GFP*-lines. The transcriptional levels of the defense-associated genes, including *PR1b*, *PR2b*, *ERF1* and *LOX*, were also down-regulated in the *PsCRN70*-transgenic lines. Our results suggest that PsCRN70 may function as a universal suppressor of the cell death induced by many elicitors, the host H_2_O_2_ accumulation and the expression of defense-associated genes, and therefore promotes pathogen infection.

## Introduction

Phytopathogens secrete a battery of effector proteins that are delivered inside host cells to promote infection [1], [2], [3]. Plants detect conserved microbial molecular signatures, termed pathogen-associated molecular patterns (PAMP), resulting in PAMP-trigger immunity (PTI). To counter PTI, pathogens evolve diverse effectors to suppress PTI and trigger susceptibility (effector-triggered susceptibility, ETS). When the effectors are recognized by the corresponding resistance (R) proteins in the host plants, effector-triggered immunity (ETI) in host cells is activated [4]. To overcome R protein-mediated immune responses, pathogens response by mutating or losing effectors, or by developing novel effectors that can suppress ETI [5]. For example, *P. infestans* secreted the effector SNE1 to suppress the R3a/Avr3a -mediated PCD [6]. In the absence of the R proteins, pathogen effectors exert virulence activity to interfere with plant immunity [1]. It has been shown that *in planta* expression of *Phytophthora* effectors, such as *P. infestans Avrblb2* [7], RxLR effector *PITG_03192* [8], *CRN8* [9], and *P. sojae Avh241* [10], enhanced susceptibility to pathogens.

Oomycete pathogens, such as *Phytophthora spp*., cause a wide variety of devastating plant diseases globally [11]. For example, *P. infestans*, a pathogen of late blight of potato and tomato, was responsible for the Irish potato famine in the mid-nineteenth century; *P. sojae* causes soybean root and stem rot and leads to substantial yield losses annually [11]. *Phytophthora* are hemibiotrophs that initiate the infection cycle as biotrophs, during which the pathogen proliferates asymptomatically in the host, and at this stage pathogens must employ efficient mechanisms to evade and suppress plant immune responses. At the later stage, hemibiotrophs switch to a necrotrophic lifestyle by killing the host plants, and this process is presumably modulated by the coordinated secretion of factors such as lytic enzymes and cell-death inducers. However, the mechanisms underlying regulation of the switch from biotrophs to necrotrophs are still largely unknown. It was reported that *P. infestans* expresses effector *SNE1* at the early infective stage to suppress cell death induced by many other effectors, which may function to maintain the biotrophic phase. A high throughput functional assay for *P. sojae* effectors revealed that *Phytophthora* pathogens may produce effectors with contrasting activity to regulate the infection process [12].

The genomes of the *Phytophthora* species contain large repertoires of RxLRs and CRNs [13], [14], [15], which are two kinds of cytoplasmic effectors. RxLR effectors are defined by a conserved N-terminal motif, which enables delivery of effector proteins inside plant cells [16], [17]. CRN effectors were first identified in *P. infestans* as proteins that resulted in a leaf-crinkling and cell-death phenotype in plants [18]. The CRN effector family showed extensive expansion in all sequenced *Phytophthora* species [13], [14]. The average expression levels of CRNs were much higher than those of the RxLR genes, indicating that CRNs may play important roles in pathogenicity [19]. Evolutionary analyses uncovered that gene duplication and fragment recombination drive functional diversity of CRN family [19]. Analogous to RxLR effectors, the N-termini of CRN effectors harbor a conserved FLAK motif, which translocates effectors inside host cells [20]. The C-terminal region of CRN proteins is diverse and controls virulence [14]. CRN1 and CRN2 were identified from *P. infestans* following an *in planta* functional screen for candidate secreted proteins, and transient expression of these two CRNs induced cell death in plants [18]. *P. infestans* CRN8, which contains a kinase domain, targets plant nucleus to induce cell death [9]. CRN63 and CRN115 were identified from *P. sojae*, and they share high sequence similarity, however, they possess contrasting biological activities on host plants, in which PsCRN63 induces cell death and PsCRN115 suppresses cell death [21].

It was previously considered that the majority of CRN effectors caused cell death, however, it has been shown that few CRN effectors can trigger cell death when expressed *in planta* [19], [22]. On the contrary, the majority of CRNs can suppress cell death triggered by PAMPs or other elicitors [19]. These observations suggested that CRN effectors may also act in the biotrophic phase, which promote infection of hemibiotrophic *Phytophthora*. Interestingly, most of the CRN effectors are localized in the plant cell nucleus when expressed *in planta* [20], [22], indicating that CRN effectors may target and perturb host nuclear processes to achieve virulence. Alteration in subcellular localization of CRN effectors blocked their cell-death-inducing activity [20], [21], which suggests that CRN effectors need target to plant cell nucleus to exert their biological functions. Recent progress showed that CRN effector family may target distinct subnuclear compartments and modify host cell signaling [23]. However, the roles of CRN effectors in virulence are still largely unknown.

Cytoplasmic effectors are translocated into host plant cells to interfere with plant immunity, and it has become an efficient strategy to study the virulence functions of effectors by expressing them *in planta* [7], [8], [9], [10]. Here we identified a CRN effector *PsCRN70* from *P. sojae*, and expressed it in a model plant *Nicotiana benthamiana* by *Agrobacterium*-mediated transient expression and stable transformation. We showed that PsCRN70 can suppress cell death induced by many cell-death inducers, such as the mouse BAX, *P. sojae* RxLR effector Avh241, CRN effector PsCRN63, necrosis-inducing protein PsojNIP, and the R3a/Avr3a. Overexpression of *PsCRN70* gene enhanced susceptibility of *N. benthamiana* to *P. parasitica*, indicating that PsCRN70 positively contributes to virulence. Our results indicated that, in addition to causing cell death *in planta*, CRN effectors may also function as a suppressor of plant cell-death and defense responses.

## Materials and Methods

### Plant material, bacterial strain and growth condition

*Nicotiana benthamiana* seeds were surface sterilized by soaking in 75% ethanol for 30 s followed by in 1.0% sodium hypochlorite for 5 min. The seeds were rinsed 5 times with sterile water. They were subsequently spread onto petri dishes containing solid 1/2 MS medium. Plates were kept at 18 °C for 4 days in the dark and then at 22 °C for 10 days in 16/8 hour light/dark cycle. The seedlings about 2–3 cm long were transferred aseptically to the glass bottles to get 7–8 young leaves. *Escherichia coli* and *Agrobacterium tumefaciens* strains carrying the disarmed Ti plasmid were routinely grown on Luria-Bertani (LB) agar or broth at 37°C and 28°C, respectively. *P. sojae* isolate P6497 and *P. parasitica* isolate Pp016 was routinely cultured on V8 medium at 28°C.

### Plasmid construction

*PsCRN70* gene (submitted to Genbank; awaiting accession numbers) lacking the predicted signal peptide was amplified using cDNA from *P. sojae* through PCR with the forward primer: 5′-acgcgtcgacATGGTGACGATCGCGTGTG-3′ and the reverse primer 5′-gctctagaTTAAGTACGACGGAGAATTC-3′. After digesting with the *Sal*I and *Xba*I restriction enzymes, the resulting PCR product was inserted into the expression vector pBinGFP2 [10]. For the PVX construct, the *PsCRN70* gene was amplified and inserted into the PVX vector pGR106 [24] using the *Sma*I and *Not*I restriction sites. The recombinant plasmids were confirmed by sequencing and introduced into *Agrobacterium* strains by electroporation.

### Generation of the *PsCRN70*-transgenic *N. benthamiana*

The *PsCRN70* gene was introduced into *N. benthamiana* using the leaf-disc transformation approach as described previously [25]. Briefly, the *Agrobacterium* EHA105 carrying the *pBinGFP:PsCRN70* construct was incubated over night at 28°C with shaking at 220 rpm to an OD_600_ of 0.4–0.6. The healthy *N. benthamiana* leaf discs were co-cultured with the *Agrobacterium* suspension for 30 min in 20 mL of liquid MS medium, and then placed on a piece of sterile filter paper and cultured on non-selective callus induce medium (CIM) which contains 1 mg/L of 6-BA at 25°C in the dark for 3 days. After 3 days the infected explants were transferred to a fresh shoot induction medium (SIM) supplemented with 1 mg/L of 6-BA, 100 mg/L of kanamycin and 500 mg/L of carbenicillin at 25°C in the light for 25–30 days for shoot regeneration. Healthy shoots that reached a length of 1–2 cm tall were excised and transferred into a jar containing the selective rooting medium (RIM) supplemented with 100 mg/L of kanamycin, 500 mg/L of carbenicillin and 0.2 mg/L of IAA for root generation. Roots were obtained after 2–3 weeks in culture and transferred to soil under growth room conditions for seed set.

### *Agrobacterium*-mediated transient expression

PVX constructs were transformed into *A. tumefaciens* strain GV3101 using the electroporation method [21]. For agroinfiltration assay, *Agrobacteria* containing the corresponding constructs were cultured in LB media containing 50 mg/mL of kanamycin at 28 °C with shaking at 220 rpm for 48 h. The culture was harvested and washed three times in 10 mM MgCl_2_, and resuspended in 10 mM MgCl_2_ to an OD_600_ of 0.3. Infiltration was performed on six-week-old *PsCRN70*-transgenic *N. benthamiana* and the *GFP*-control plants. Symptom development was monitored and photographs were taken 4–6 days post infiltration. The experiments were repeated at least three times.

### *Phytophthora parasitica* inoculation assay

We used two approaches, transient and stable expression, to evaluate the role of PsCRN70 in suppression of plant immunity. For transient expression approach, we expressed the *PsCRN70* and *GFP* in *N. benthamiana* leaves using agroinfiltration method, and inoculated with *P. parasitica* zoospores 2 days post infiltration. Briefly, the *P. parasitica* zoospores were prepared as described previously [26] and *N. benthamiana* leaves were detached and placed in a plastic tray, then each leaf was inoculated with 20 µL of zoospore suspensions with a concentration of 100 zoospores per microliter on the abaxial surface of the leaf. Phenotype was monitored within 72 h, and photographs were taken 36 hours post-inoculation.

For the stable *PsCRN70*-transgenic *N. benthamiana*, we used the detached leaves and the whole seedlings of 5-week old to evaluate the role of PsCRN70 in plant defense. The resistant levels of the whole transgenic plants were assessed using the root-dip inoculation assay. Twenty plants for each T2 transgenic line (#1, #3, #4 and #12) were inoculated with the *P. parasitica* zoospore suspensions, and the *GFP*-transgenic lines were used as the control. The inoculated plants were kept in a moist chamber, and the disease progression was monitored within 10 days. At least 3 independent experiments were performed for this assay. Duncan's multiple range test (SPSS Statistical software version 16.0) was used for statistical analysis (P<0.01).

### RNA extraction and quantitative RT-PCR

Total RNA was extracted from *N. benthamiana* leaves using the RNeasy Mini Kit (Qiagen) according to the manufacturer's instructions. The cDNA was generated using the PrimeScript RT reagent Kit (Takara). Real-time quantitative PCR was performed in 20- µL reactions including 20 ng of cDNA, 0.2 µM gene-specific primers, 0.4 ul ROX Reference Dye, 10 µL of SYBR Premix Ex Taq (Takara), and 6.8 µL of deionized water. PCR was performed on an ABI PRISM 7300 Fast Real-Time PCR System (Applied Biosystems) under the following conditions: 95°C for 30 s, 40 cycles of 95°C for 5 s and 60°C for 31 s to calculate cycle threshold values, followed by a dissociation program of 95°C for 15 s, 60°C for 1 min, and 95°C for 15 s to obtain the melt curves. The *N. benthamiana EF1α* gene was used as the internal reference gene for calculating relative transcript levels. An equal volume of cDNA was used for gene analysis expression of defense-related genes, using specific primers *PR1b* 5′- GTGGACACTATACTCAGGTG-3′/5′-TCCAACTTGGAATCAAAGGG-3′, *PR2b* 5′-AGGTGTTTGCTATGGAATGC-3′/5′-TCTGTACCCACCATCTTGC-3′, *ERF1* 5′-GCTCTTAACGTCGGATGGTC-3′/5′-AGCCAAACCCTAGCTCCATT-3′, *LOX* 5′-AAAACCTATGCCTCAAGAAC-3′/5′-ACTGCTGCATAGGCTTTGG-3′ and *EF1α* 5′-AGAGGCCCTCAGACAAAC-3′/5′-TAGGTCCAAAGGTCACAA-3′. The induction ratio of treatment/control was then calculated by the 2^-ΔΔCT^ approach. Student t-test was used for statistical analysis.

### Confocal microscopy

To observe subcellular localization of PsCRN70, the transgenic *N. benthamiana* leaves were cut into small squares. The leaf squares or plant roots were then immersed into PBS buffer containing 5 µg/mL DAPI for staining of the nuclei for 5 min. Sildes carrying the samples were observed with a Zeiss LSM 710 confocal laser scanning microscope (CLSM). The excitation wavelength used for GFP was 488 nm and 405 nm for DAPI. The *GFP*-transgenic *N. benthamiana* leaves were used as the control. Images were progressed using the Zeiss 710 CLSM and Adobe Photoshop software packages.

### Protein extraction and Western blot analyses

The *N. benthamiana* leaf tissues were ground in liquid nitrogen. The ground materials were mixed with protein extraction buffer [50 mM Hepes, 150 mM KCL, 1 mM EDTA, 0.1% Triton X-100, adjust pH to 7.5 with KOH] supplemented with 1 mM DTT, and 1× protease inhibitor mixture (Roche). Crude plant protein extracts were collected by centrifuging at 12,000×g at 4 °C for 15 min. Protein extracts were loaded on 12% SDS- polyacrylamide gels and protein gel blot analyses were performed as reported previously [10]. Briefly, proteins were transferred from the gel to an Immobi-lon-P^SQ^ polyvinylidene difluoride membrane after electrophoresis. The membranes were washed in PBST (PBS with 0.1 Tween 20) for 2 min and then blocked in PBSTM (PBS with 0.1% Tween 20 and 5% non-fat dry milk) for 1 h. Mouse monoclonal antibody against GFP or HA was added into PBSTM and incubated for 90 min, followed by washing with PBST for three times. The membranes were then incubated in PBSTM with a goat anti-mouse IRDye 800CW (Li-Cor) for 40 min. The membranes were washed for three times with PBST and then visualized using a LI-COR Odyssey scanner with excitation 700 and 800 nm.

### DAB staining

H_2_O_2_ was visualized using the 3,3-diaminobenzi-dine (DAB) (Sigma) staining approach as described previously [27]. Leaves were inoculated with *P. parasitica* as described above; infected leaves 12 hours post inoculation were soaked in the DAB aqueous solution at 1 mg/ml and maintained at 25°C for 8 h. Leaf sections were cleared by boiling in 95% ethanol for 15 min, bleaching solution was replaced and leaves were incubated until the chlorophyll was completely bleached. DAB-staining experiments were independently repeated at least three times. Duncan's multiple range test were used for statistical analysis (P<0.01).

## Results

### Generation of the *PsCRN70*-transgenic *N. benthamiana*

**Figure 1:**
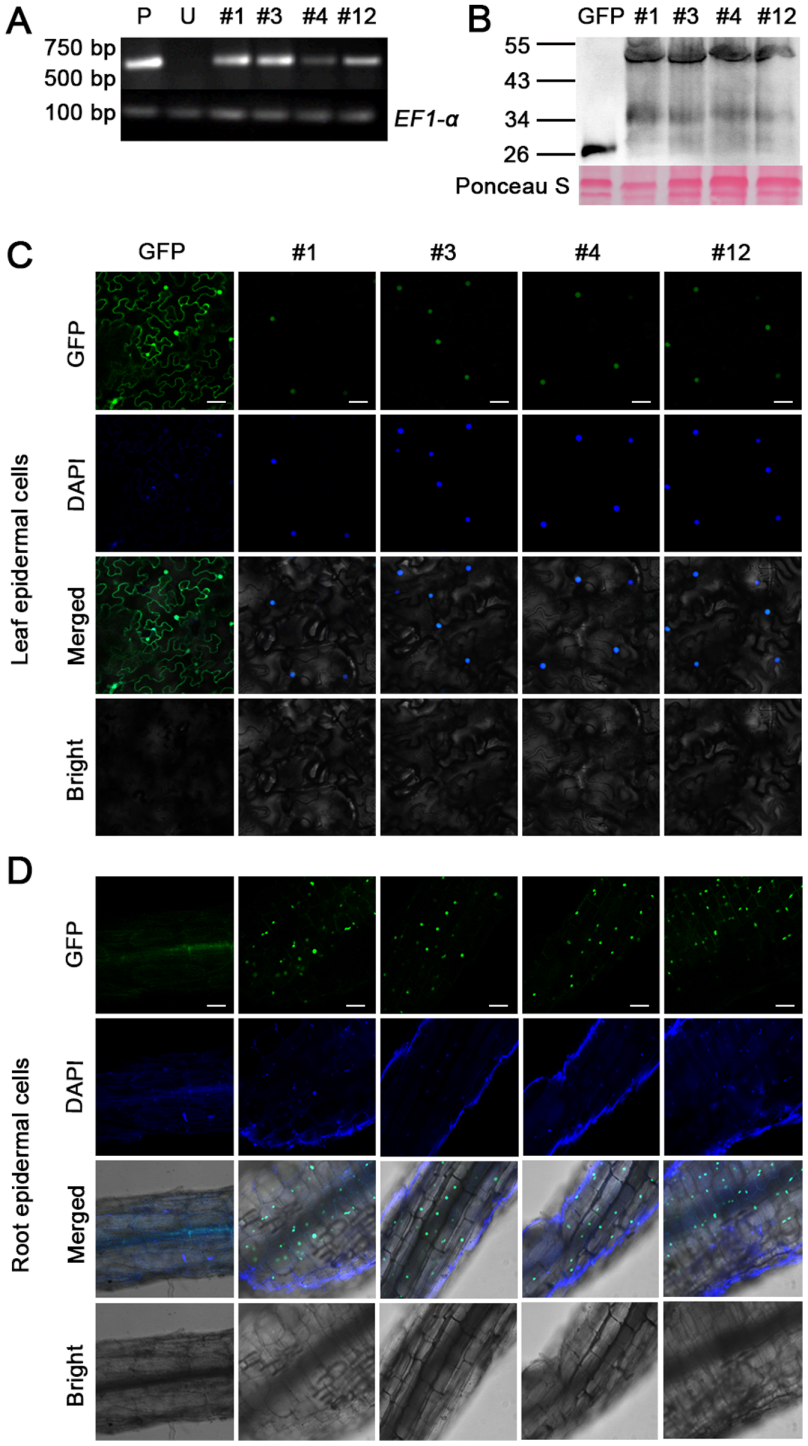
Characterizations of the *PsCRN70-*transgenic *N. benthamiana*. A. RT-PCR analysis of *PsCRN70* expression in independent transgenic lines. #1, #3, #4 and #12, four independent T1 transgenic lines; P, *pBinGFP:PsCRN70* plasmid as a positive control; U, untransformed plant as a negative control. The upper panel represents the 624 bp fragment of *PsCRN70* gene and the lower panel represents the 100 bp fragment of *EF1α* gene as the control. B. Western blot analysis of expression of GFP: PsCRN70 fusion protein in transgenic *N. benthamiana* using monoclonal antibody against GFP. Subcellular localization of PsCRN70 in the transgenic *N. benthamiana* leaf tissues (C) and roots (D). The pictures were taken using a confocal microscope. The scale bar indicates 50 µm.

Introduction of *PsCRN70* gene into *N. benthamiana* were achieved by *Agrobacterium*-mediated leaf disc transformation. Integration of the *PsCRN70* and *GFP* transgenes were confirmed by PCR analysis. Thirty independent transgenic lines (T0) including ten *GFP*-transgenic plants and twenty *PsCRN70*-transgenic plants were obtained, and fifteen of them were randomly selected and self-pollinated to produce T1 lines. RT-PCR analysis confirmed that the *GFP:PsCRN70* fusion genes were highly expressed in four (#1, #3, #4 and #12) independent T1 transgenic lines (Figure 1A). The seedlings of the transgenic progenies (T1 or T2 generations) were screened by checking fluorescent signal of GFP for further analyses. We confirmed the expression of GFP:PsCRN70 fusion protein using Western blot in transgenic lines, and the result showed that the fusion protein was correctly expressed (Figure 1B). Observation of the GFP fluorescence also validated the expression of the transgenes (Figure 1C). No visible differences in plant growth and other phenotypes were observed between the *PsCRN70*-transgenic plants and the *GFP*-transgenic lines, indicating that expression of *PsCRN70* did not affect the development of *N. benthamiana* under normal growth conditions.

It was reported that the majority of CRN proteins are localized in the plant cell nuclei [20], [23]. To test the subcellular localization of the target proteins, we first analyzed whether PsCRN70 harbors an NLS using the cNLS Mapper software [28], and the result showed that PsCRN70 contained a typical NLS (YLARRKKRKEE) in the C-terminal region, indicating that PsCRN70 may also target the plant cell nucleus. We then observed the distribution of the GFP fluorescent signal in the four T2 transgenic plants (#1, #3, #4 and #12) and the *GFP*-control lines under a confocal microscope using the leaf and root tissues, respectively. The GFP fluorescent signal in the *PsCRN70*-transgenic lines was dominantly distributed in the nucleus of the leaf epidermal cells (Figure 1C). The fluorescent signal in the root epidermal cells of the *PsCRN70*-transgenic *N. benthamiana* was also distributed mainly in the plant nucleus, which was further confirmed by co-localization with DAPI staining (Figure 1D). In contrast, the fluorescent signal in the *GFP*-transgenic lines was equally distributed in the cytoplasm and nucleus of the leaf and root epidermal cells (Figure 1D). Thus, we concluded that PsCRN70 is a plant nucleus-localized protein.

### Expression of the *PsCRN70* in *N. benthamiana* enhance susceptibility to *P. parasitica*

**Figure 2:**
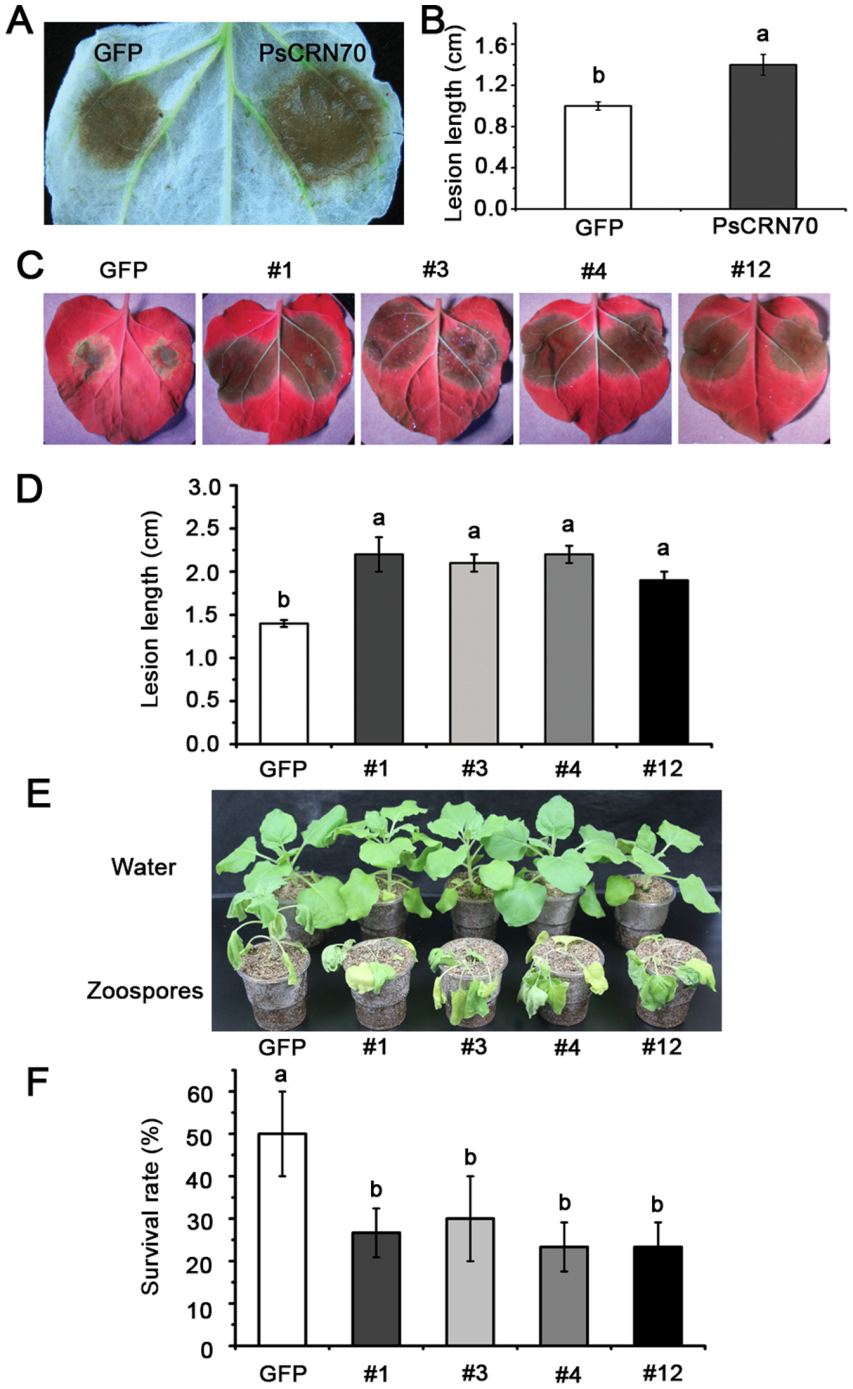
Suppression of the plant resistance by PsCRN70. A. Lesions on the *N*. *benthamiana* leaves expressing *PsCRN70* or *GFP*. A decolorized infected leaf was photographed 4 dpi. The experiments were repeated three times and shown with a representative image. B. Average lesion diameters on *N*. *benthamiana* leaves expressing the indicated genes inoculated with *P. parasitica*. Averages were calculated from four lesions per construct. Error bars represent standard errors. Different letters at the top of the columns indicate significant differences (P<0.01, Duncan's multiple range test). C. Phenotypes of the *PsCRN70*- and *GFP-*transgenic lines inoculated with *P. parasitica*. Detached leaves from five-weak old seedlings (T1 generation) of transgenic plants (#1, #3, #4, #12) were inoculated with the *P. parasitica* zoospores. Photographs were taken 36 hpi under a UV lamp. D. Lesion diameters on inoculated leaves measured 36 hpi. The experiments were repeated four times in all transgenic lines with similar results. Bars represent the standard deviation (SD). Different letters at the top of the columns indicate significant differences (P<0.01, Duncan's multiple range test). E. Phenotypes of the *PsCRN70*-transgenic plants inoculated with *P. parasitica* zoospores. The transgenic lines were inoculated with zoospores using the root dip method. Mock-inoculated seedlings were inoculated with sterile distilled water. Photographs were taken 4 days post inoculation. F. Survival rates of the transgenic plants inoculated with *P. parasitica* zoospores. The rates were measured at 4 dpi. The experiments were repeated four times in all transgenic lines with similar results. Twenty plants were used for each treatment in each experiment. Bars represent the standard deviation (SD). Different letters at the top of the columns indicate significant differences (P<0.01, Duncan's multiple range test).

To elucidate roles of PsCRN70 in *N. benthamiana* resistance, we constructed *PVX:PsCRN70* and *PVX:GFP*, and then transiently expressed them in *N. benthamiana* leaves. Two days post-infiltration, the infiltrated leaves were challenged with the *P. parasitica* zoospores. The detached leaf tissues expressing the *PsCRN70* developed larger lesion compared to those expressing the *GFP* control (Figure 2A). On leaf areas expressing the *GFP* gene, the average diameter of the lesions was ∼1.0 cm 36 hours post-inoculation; however, on areas infiltrated with *PsCRN70*, the average lesion diameter was ∼1.4 cm (Figure 2B). Thus, transient expression of the *PsCRN70* in *N. benthamiana* reduced its resistance to *P. parasitica*.

To validate the above observations, we also did inoculation assays of leaves and roots using stable *PsCRN70-*transgenic plants of T1 generation. The average lesion diameters were significantly larger in the *PsCRN70-*transgenic leaves than that in the *GFP*-transgenic leaves (Figure 2C, D). Root inoculations resulted in both *PsCRN70*- and the *GFP*-transgenic plants (T2 generation) displaying symptoms of wilting and stunting (Figure 2E). However, the survival rate of the *PsCRN70*-transgenic plants was significantly lower than the *GFP*-transgenic plants (Figure 2F). Taken together, these results showed that PsCRN70 enhanced susceptibility of *N. benthamiana* to *P. parasitica* infection.

To determine whether expression of *PsCRN70* enhanced susceptibility of *N. benthamiana* to nonhost pathogen, we inoculated the transgenic *N. benthamiana* leaves (T2 generation) with *P. sojae* mycelial plugs. No obvious infection was found in both *GFP*- and *PsCRN70*-transgenic plants 5 days post inoculation (Figure S1), and this indicated that expression of *PsCRN70* in *N. benthamiana* did not affect its nonhost resistance to oomycete pathogen *P. sojae*.

### PsCRN70 suppresses cell death in *N. benthamiana*

**Figure 3:**
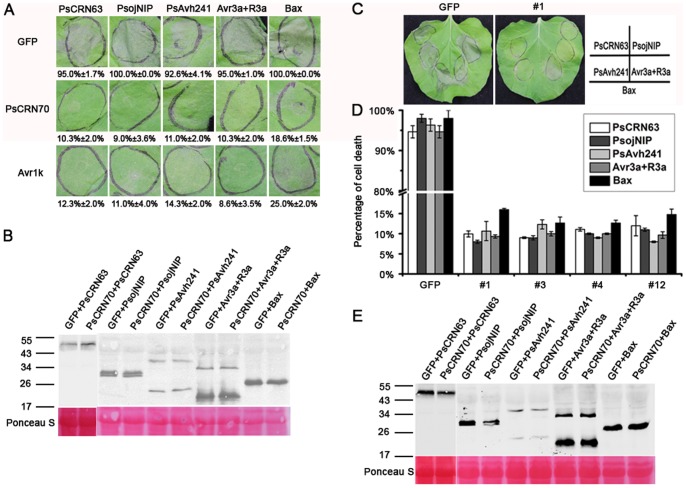
Suppression of cell death by PsCRN70. A. Transient expression assay of *PsCRN70* in *N. benthamiana* leaves. Agroinfiltration sites in each *N. benthamiana* leaf expressing *GFP* (upper panel, a negative control), *Avr1k* (lower panel, a positive control), and *PsCRN70* (middle panel) were challenged after 12 h with *A. tumefaciens* carrying the indicated cell death-inducers. Photographs were taken 5 d after cell death inducer infiltration. The data is the percentage of cell death sites from 8 infiltrated leaves based on three independent experiments. B. Western blot analysis of expression of the cell-death inducers in *N. benthamiana* leaves transiently co-expressed with *GFP* or *PsCRN70*. Antibody against the HA-epitope tag was used to detect the expression of *PsCRN63*, *PsojNIP*, *PsAvh241*, *Avr3a* and *Bax* when they were co-expressed with *GFP* or *PsCRN70*. C. Cell-death-suppression assay on leaves of the stable *PsCRN70*-transgenic *N. benthamiana*. D. The percentage of cell death sites. The percentages of cell death sites on the *PsCRN70*-transgenic lines were scored from 8 infiltrated leaves based on three independent experiments. E. Western blot analysis of expression of the cell-death inducers in leaves of *PsCRN70*-transgenic *N. benthamiana* two days post infiltration. The *PsCRN70*-transgenic lines (T2 generation of #1 was shown as an example) were infiltrated with *A. tumefaciens* containing the *PsCRN63*, *PsojNIP*, *PsAvh241*, *Avr3a*/*R3a* and *Bax*; the *GFP*-transgenic plant was used as the control. Photographs were taken 5 d after cell death inducer infiltration.

To characterize how PsCRN70 contributes to virulence, we tested its cell-death-manipulation activity using the *Agrobacterium*-mediated transient expression in *N. benthamiana*. This approach has been widely used for analyzing cell-death manipulation [14], [21]. The cell-death inducing proteins included the mouse BAX [29], the *P. sojae* necrosis-inducing protein PsojNIP [30], the RxLR effector PsAvh241 [10], the CRN effector PsCRN63 [21] and the R3a/Avr3a [31]. *P. sojae* effector Avr1k [32] can inhibit cell death induced by all the above elicitors and was used as a positive control. As expected, no cell death phenotypes were observed in the *Avr1k*-infiltrated leaves (Figure 3A). Expression of the *PsCRN70* gene also blocked cell death triggered by these elicitors 5 days post-infiltration. As a negative control, expression of the *GFP* gene did not suppress the cell death (Figure 3A). Western blot result showed that the cell-death inducers were expressed at similar levels when co-expressed with *GFP* control or *PsCRN70*, indicating that suppression of cell death was due to expression of *PsCRN70*. We further validated the results in the stable *PsCRN70*-transgenic *N. benthamiana*. In the transgenic line expressing *GFP*, cell death occurred at 5 days after infiltration with the above tested elicitors. However, cell death symptoms were only occasionally and weakly observed in the four *PsCRN70*-transgenic lines under the same conditions (Figure 3C, D). Western blot results also showed that the cell-death inducers were expressed at similar levels in *PsCRN70*-transgenic and *GFP*-lines (Figure 1E). Collectively, these results suggested that PsCRN70 may function as a broad cell death-suppressor to manipulate the plant immunity.

### Expression of the *PsCRN70* impairs the H_2_O_2_ accumulation in *N. benthamiana*

**Figure 4:**
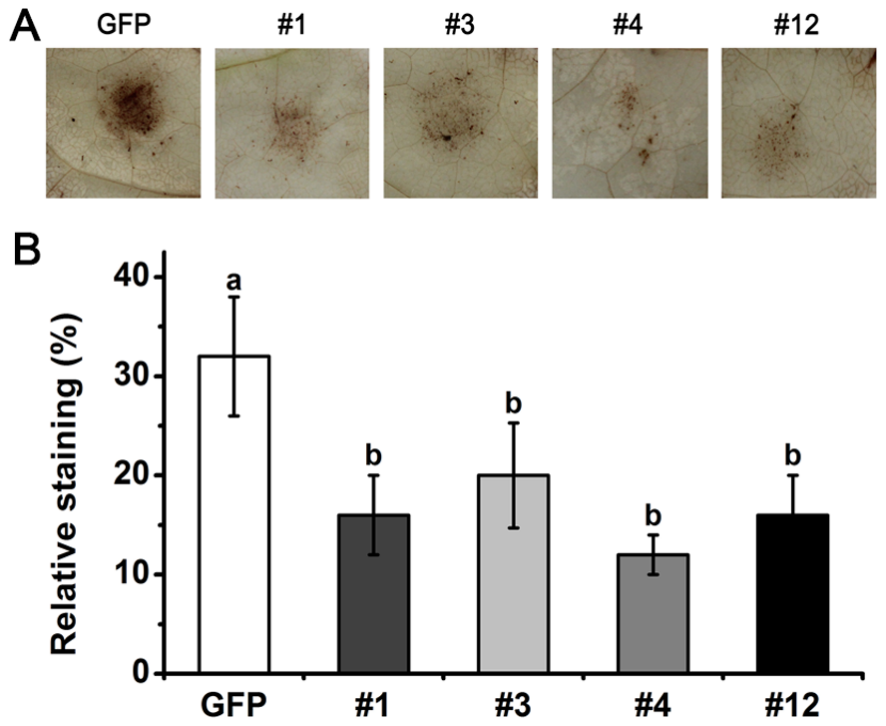
Suppression of the H_2_O_2_ accumulation in *N. benthamiana* by PsCRN70. A. DAB staining of the *P. parasitica*-inoculated *N. benthamiana* leaves. The H_2_O_2_ accumulation in the *PsCRN70*- and *GFP*- transgenic leaves were detected using DAB staining at 12 hpi. Photographs were taken after de-colorization of leaves with ethanol. B. The relative levels of DAB staining. The data were calculated by a combination of Photoshop and Quantity One for H_2_O_2_ accumulation in the indicated transgenic lines. The experiments were repeated three times in all transgenic lines with similar results. Four leaves were used for each treatment in each experiment. Bars represent the standard deviation (SD). Different letters at the top of the columns indicate significant differences (P<0.01, Duncan's multiple range test).

H_2_O_2_, a kind of reactive oxygen species (ROS), plays an important role in plant defense responses [27]. To examine the role of PsCRN70 on the plant H_2_O_2_ accumulation, the H_2_O_2_ levels in the transgenic plants were detected at the early infective stages of *P. parasitica* using the DAB staining method. Weak staining was observed in the infected area of the *PsCRN70*-transgenic leaves (Figure 4A). The relative staining was significantly lower in the *PsCRN70*-transgenic *N. bethamiana* leaves compared to that in the *GFP*-control plants (Figure 4B). This result suggested that PsCRN70 may promote *Phytophthora* infection by suppressing the H_2_O_2_ accumulation.

### Expressions of the *PsCRN70* reduces the expressional levels of the plant defense-associated genes

**Figure 5:**
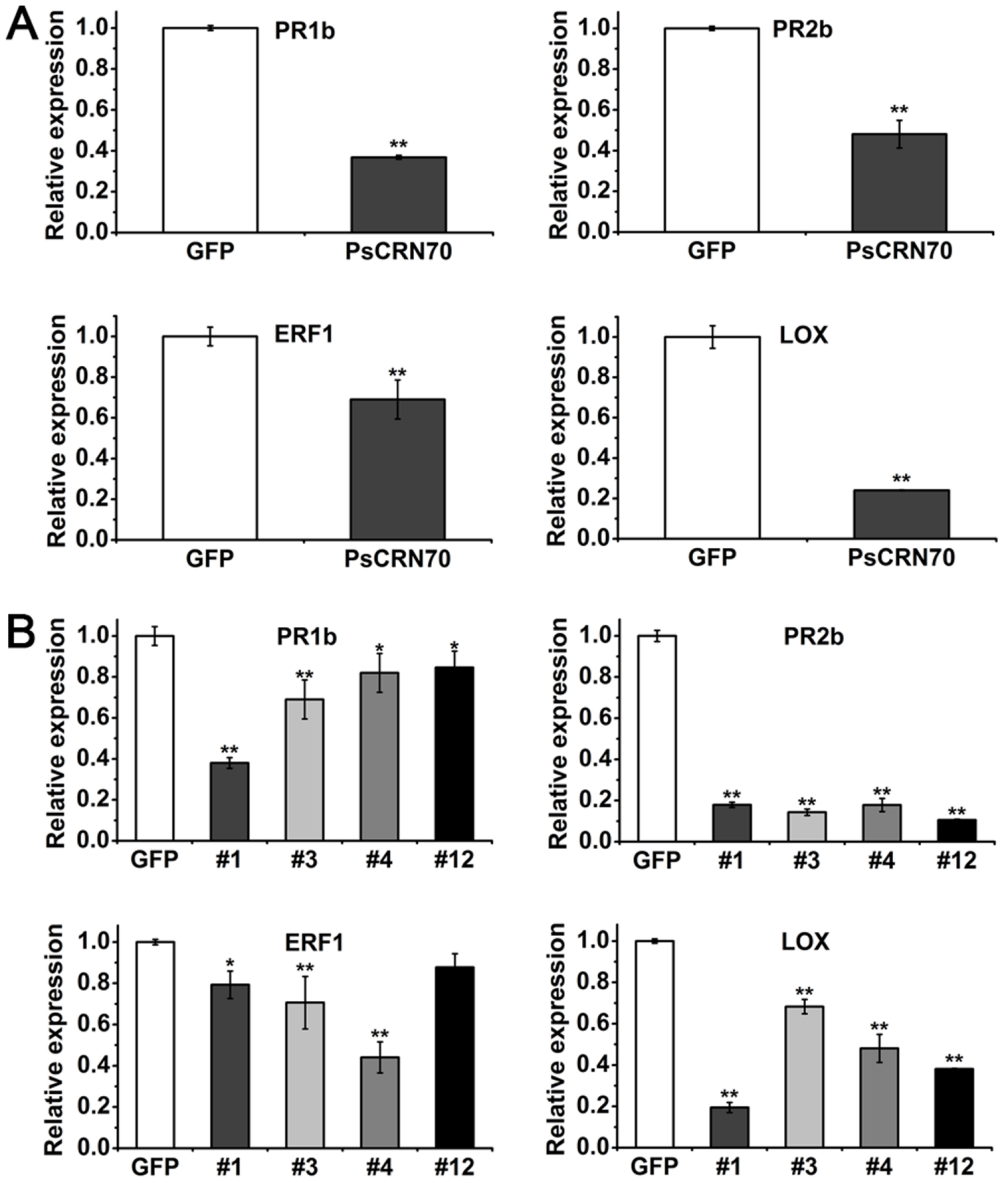
Down-regulation of the defense-associated genes in *N. benthamiana* by PsCRN70. A. Relative expression levels of the *PR1b*, *PR2b*, *ERF1* and *LOX* genes. The total RNA was extracted from the leaf tissues that transiently expressing *PsCRN70* and *GFP*, respectively, and the expression levels of the indicated genes were measured using qRT-PCR. B. Relative expression levels of the indicated genes in the stable transgenic *N. benthamiana*. The *N. benthamiana* leaves 36 hpi with *P. parasitica* zoospores were collected, and the gene expression levels were measured by qRT-PCR. The gene expression levels were normalized to the *EF1α* gene. Bars represent the standard deviation (SD), with significant difference (** for P<0.01 and * for P<0.05, Student's *t*-test).

To further assess the role of PsCRN70 in plant defense responses, we examined the transcriptional levels of the defense-associated genes in plants, including *PR1a* (*Pathogenesis-related protein*), *PR2b*, *ERF1* (*Ethylene response factor 1*) and *LOX* (*Lipoxygenase*) genes, among which, the *PR1a* and *PR2b* genes are markers in the salicylate-mediated signaling pathway [33]; the *ERF1* is a marker in the ethylene-mediated signaling pathway [34]; and the *LOX* is a marker in the jasmonate-mediated signaling pathway [35]. These genes are all involved in downstream of the defense signaling pathways. Expressional levels of all the four tested genes were significantly repressed in the *N. benthamiana* leaves transiently expressing *PsCRN70* compared to that in leaves expressing *GFP* (Figure 5A). The expression levels of the four genes also exhibited significant reduction in the stable *PsCRN70*-transgenic lines when challenged with *P. parasitica* zoospores (Figure 5B). These results suggest that PsCRN70 may repress the expression of the defense-associated genes in plants.

## Discussion

*Phytophthora* pathogens encode a large number of RxLR and CRN effectors [13], [14], [15], however, virulence functions of CRN effectors are largely unknown. Overexpression of the *PsCRN70* in *N. benthamiana* enhanced susceptibility to *P. parasitica*, indicating that PsCRN70 contributes to pathogen virulence. DAB staining results showed that the H_2_O_2_ accumulation in the *PsCRN70*-transgenic plants were significantly lower than that in the control lines, indicating that PsCRN70 can promote *Phytophthora* infection by reducing H_2_O_2_ levels in plants. The role of H_2_O_2_ in plant defense responses has been extensively studied [36]. It has been adopted by many pathogens to promote infection by regulating H_2_O_2_ production in plants [1], [37]. For example, *Ustilago maydis* secretes the effector Pep1 into the apoplast to suppress the H_2_O_2_ production, resulting in suppression of plant immunity [37]. Quantitative RT-PCR results showed that the marker genes from different hormone signaling pathways were significantly down-regulated in the *PsCRN70*-transgenic *N. benthamiana* compared to the control, which further confirmed that PsCRN70 significantly reduced plant defense responses. SA and JA signaling pathways usually act antagonistically in plant defense. We showed that PsCRN70 may suppress both pathways, indicating the effector protein exhibits broad suppression activities.

It was originally surmised that the expression of CRN effectors triggered cell death [18]. However, more recent studies suggest that only a few CRNs induce necrosis [19], [22]. Our results showed that PsCRN70 can suppress cell death induced by many elicitors including the mouse BAX, *P. sojae* RxLR effector Avh241, CRN effector PsCRN63, necrosis-inducing protein PsojNIP, PCD triggered by the resistance protein R3a and the avirulence protein Avr3a. These results indicate that, similar to the SNE1 from *P. infestans* [6] and Avr1k [32] from *P. sojae*, PsCRN70 may function as a broad cell-death suppressor to promote *P. sojae* infection. These broad acting cell-death suppressor proteins will be useful tools in identifying the components of protein regulatory networks in immune signaling and cell death pathways.

It has been reported that the majority of CRN effectors are localized in the plant cell nuclei [20]. PsCRN70 is also located in the plant cell nucleus, indicating that function by members of the CRN effector family may function by manipulating the host nuclear processes to suppress the plant immune signaling. Subcellular localization of several cell-death-inducing CRN effectors demonstrated that they target distinct subnuclear compartments [23], indicating that CRNs may interfere with diverse nuclear targets. Alteration of nuclear targeting signals in several CRNs blocked their cell death-inducing activity [20], [21]. However, it was unclear whether the nuclear subcellular localization of CRNs can account for the increase in effector virulence due to the expression of these proteins. In conclusion, we showed that PsCRN70 may function as a broad cell-death suppressor to promote *Phytophthora* infection, providing insight into the role of CRN effectors in pathogenicity.

## Supporting Information

Figure S1 **Phenotypes of the** ***PsCRN70*** **-transgenic plants inoculated with** ***P. sojae*** **mycelial plugs.** Photographs were taken 5 days post inoculation.
(TIF)
