# Supplementary figures and images for: Phytophthora sojae Effector PsCRN70 Suppresses Plant Defenses in Nicotiana benthamiana
Source: PLoS One. 2014 May 23;9(5):e98114. doi: 10.1371/journal.pone.0098114 (PMC4032284; doi:10.1371/journal.pone.0098114)

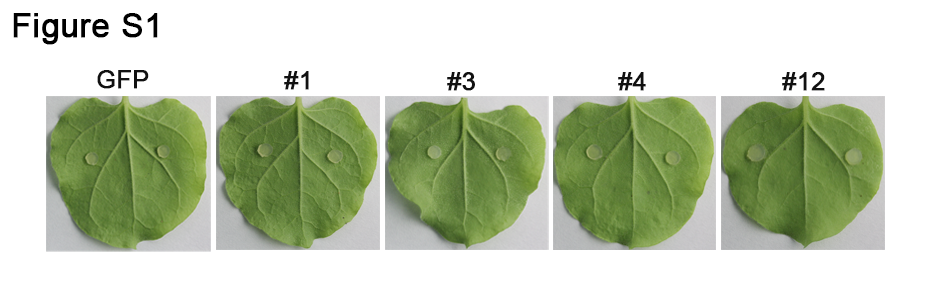

Supplement: Figure S1 — Phenotypes of the PsCRN70 -transgenic plants inoculated with P. sojae mycelial plugs. Photographs were taken 5 days post inoculation. (TIF) [file pone.0098114.s001.tif]
